# Innovation leadership perspective in public health

**DOI:** 10.1093/eurpub/ckae217

**Published:** 2025-03-25

**Authors:** Moredreck Chibi, Gauden Galea

**Affiliations:** Special Initiative for NCDs and Innovation, World Health Organisation Regional Office for Europe, 2100 Copenhagen, Denmark; Special Initiative for NCDs and Innovation, World Health Organisation Regional Office for Europe, 2100 Copenhagen, Denmark

Innovation leadership in public health has become increasingly crucial in an era of complex global health challenges and rapid technological advancements. This commentary explores the concept of innovation leadership, its importance in public health, and the key factors that influence its effectiveness. As the world grapples with unprecedented health crises and the need for sustainable solutions, the demand for innovation in public health has intensified. However, the sector often struggles to fully embrace innovation due to various factors, including lack of agility, complex regulatory pathways, incompatible organizational culture, and deficient change management skills [1]. At the heart of these challenges lies the critical need for innovation leadership to create an enabling environment that spurs innovation to accelerate health improvements and sustainable public health impact.

Innovation leadership in public health, a concept rooted in David Gliddon’s 2006 work, can be defined as the ability to envision, initiate, and guide novel approaches to addressing population health challenges through creative problem-solving, calculated risk-taking, and fostering a culture of continuous improvement [2]. This leadership style, strongly associated with transformational leadership, requires a unique blend of scientific expertise, political acumen, and the ability to inspire multidisciplinary teams. Public health leaders face distinct challenges, including navigating complex bureaucratic systems, balancing evidence-based practices with novel approaches, maintaining public trust while driving change, and operating within financial constraints and human resource limitations. To appreciate what Innovation leadership entails, one must first understand the dynamics of innovation. A study involving over a hundred thousand people from 84 countries and various companies categorized innovators into four types: generators, conceptualizers, optimizers, and implementers, whose complementary roles constitute the end-to-end innovation cycle [3]. The authors argued that many industry innovation barriers arise from not recognizing these four styles, from not providing the right incentives to not building teams that have the right mix of all four styles.

Adapting private sector innovation models to public health presents unique challenges and opportunities. The World Health Organization’s (WHO) development of the STEPwise approach to noncommunicable diseases (NCDs) risk factor surveillance (STEPS) serves as a prime example [4]. In applying the innovation cycle to STEPS, several barriers were encountered, including the public health sector’s focus on population-level outcomes rather than profit margins, which required a shift in value definition and measurement, and the complex stakeholder environment in public health that necessitated a more collaborative approach to innovation. Resource constraints in many public health systems limit the ability to rapidly prototype and iterate solutions. Therefore, capacity building and knowledge transfer for sustainable implementation, developing standardized yet flexible protocols to balance global comparability with local adaptability, and fostering partnerships to overcome resource limitations and enhance knowledge sharing should be prioritized. The STEPS approach supports monitoring a few modifiable NCD risk factors that reflect a large part of the future NCDs burden to identify interventions considered effective. This adaptation demonstrates how public health organizations can effectively translate private sector innovation models to address complex global health challenges while accounting for the unique constraints and objectives of the public health sector.

Traction in innovation is gained by first recognizing it as a source of value generation followed by rallying commitment of transformational leadership either at the global, regional, national, or organizational level for support. This is why innovation practitioners always appeal to governments to provide leadership through deliberate actions that strengthen innovation ecosystems. Such actions range from setting up institutional governance mechanisms, adopting innovation-friendly policies, and providing fiscal and non-fiscal incentives to spur innovation [5]. As a case in point, countries that enjoyed the benefits of technological developments for Coronavirus disease (COVID-19) relied heavily on transformational leadership that spearheaded laying the foundation of technological infrastructures, policies, and incentive mechanisms, which made it possible to spin off various innovations that contributed to the pandemic response [6].

Innovation has been identified as critical toward accelerating universal health coverage and reaching the Sustainable Development Goals (SDGs). In view of this, the United Nations (UN) and other UN specialized agencies like WHO are adopting innovation strategies [7], policies, and frameworks [8] that define a new trajectory to accelerate the delivery of their mandates, while putting sound leadership at the center of shaping and driving the innovation agenda. For instance, the Sustainable Development Goal 3 Global Action Plan (SDG3 GAP) [9] recognizes the importance of leadership in the coordination of stakeholders to align the global research system, elevate national research priorities and identify some catalytic actions and evidence that help determine factors for successful scale-up of innovations.

At the microeconomic level, the study done by Deloitte in 2021 [10] unearthed critical insights into how corporate innovation succeeds in the present day. Through a survey and interviews of more than 400 business, technology, and innovation leaders across various sectors in the USA, the study analysis demonstrated that organizations with a robust executive level commitment to, and understanding of, innovation are more likely to achieve sustainable impact. The critical success factors were the ability of leadership to develop a vision of innovation, value creation, the corporate role in creating an entrepreneurial culture, providing financial resources, and designing the metrics that assess progress and impact.

Given the above, there is an urgent need to empower public sector strategic managers, especially in public health, to be competent innovation leaders that unlock opportunities presented by innovation and lead anticipatory institutions. Vision, foresight, inspiration, values, disruption, empowerment, motivation, engagement, influence, communication, problem-solving, transparency, adaptation, empathy, and continuous learning, among others, are foundational competencies that leaders should build to enable the public sector to be innovative and adopt innovations for sustainable impact. These competencies are crucial for fostering cross-sector collaboration, facilitating partnerships between public health agencies, private sector entities, and community organizations, leading to more comprehensive and effective health interventions.

The success of innovation leadership is measured primarily by considering four key attributes. These include the ability to (i) set the direction with a focused and compelling purpose that is translated into targeted outcomes, which align with the goals of the organization and key stakeholders; (ii) facilitate relationships among the team members and across the organization through fostering mutual trust and respect, open and effective communication, shared accountability for results and productive patterns of collaboration; (iii) develop know-how and capabilities that the team requires to undertake the task at hand, encourage collaborative orientation to teamwork, and uphold high personal standards of performance; and (iv) driving for results enjoyed through shared ownership while fostering accountability and learning. While these attributes are crucial for innovation leadership, they are not necessarily unique to this leadership style and overlap with other effective leadership approaches, including transformational leadership. However, what sets innovation leadership apart is the specific application of these attributes in the context of driving and managing innovation, particularly in public health.

In conclusion, aspiring leaders who want to drive the innovation agenda to create societal value should be capacitated to enhance their capabilities to lead people and deliver innovation strategies, programs, and services for sustainable health impact. Innovation leadership competencies should be built on aspiring leaders’ unique talents and skills. Authentic innovation leadership is demonstrated in one’s ability to function as a role model, an effective team leader, and a true agent for organizational change. Additionally, values-driven decision-making plays a significant role in innovation leadership. It involves ethical considerations, promoting equity and inclusivity, prioritizing sustainability, maintaining transparency, and ensuring accountability. By incorporating these principles, innovation leaders in public health can ensure that their efforts not only drive progress but also uphold the fundamental principles of public health and societal well-being.

Conflict of interest: None declared.
